# Self-Locking in Collapsed Carbon Nanotube Stacks via Molecular Dynamics

**DOI:** 10.3390/ijms251910635

**Published:** 2024-10-02

**Authors:** Andrea Pedrielli, Simone Taioli, Nicola Maria Pugno

**Affiliations:** 1 Materials and Topologies for Sensor & Devices (MTSD), Sensors and Devices Center, Fondazione Bruno Kessler, 38123 Trento, Italy; pedrielli@fbk.eu; 2 European Centre for Theoretical Studies in Nuclear Physics and Related Areas (ECT*), Fondazione Bruno Kessler, 38123 Trento, Italy; taioli@ectstar.eu; 3 Trento Institute for Fundamental Physics and Applications (TIFPA-INFN), 38123 Trento, Italy; 4 Laboratory for Bioinspired, Bionic, Nano, Meta Materials & Mechanics, Department of Civil, Environmental and Mechanical Engineering, University of Trento, 38123 Trento, Italy; 5 School of Engineering and Materials Science, Queen Mary University of London, Mile End Road, London E1 4NS, UK

**Keywords:** carbon nanotube stacks, mechanical properties, self-locking, molecular dynamics

## Abstract

Self-locking structures are often studied in macroscopic energy absorbers, but the concept of self-locking can also be effectively applied at the nanoscale. In particular, we can engineer self-locking mechanisms at the molecular level through careful shape selection or chemical functionalisation. The present work focuses on the use of collapsed carbon nanotubes (CNTs) as self-locking elements. We start by inserting a thin CNT into each of the two lobes of a collapsed larger CNT. We aim to create a system that utilises the unique properties of CNTs to achieve stable configurations and enhanced energy absorption capabilities at the nanoscale. We used molecular dynamics simulations to investigate the mechanical properties of periodic systems realised with such units. This approach extends the application of self-locking mechanisms and opens up new possibilities for the development of advanced materials and devices.

## 1. Introduction

**Figure 1:**
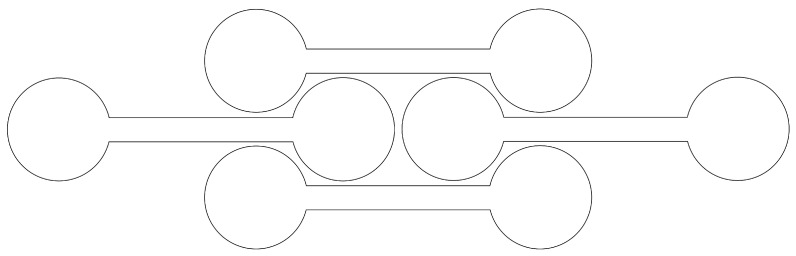
Cross-sectional view of a self-locking arrangement of dumbbell-shaped tubes.

Thin-walled round tubes are commonly used as energy absorbers on a macroscopic scale [1,2,3]. However, the structures based on stacks of round tubes are prone to splashing without adding internal connections or lateral restraints. Since the insertion of inter-tubes or lateral restraints may be undesirable or cumbersome to implement, various strategies have been proposed to prevent splashing. One strategy to avoid lateral splashing is based on elements that interlock to form self-locking structures. This concept has already been applied, for example, in developing of energy absorbers on a macroscopic scale [4]. In this context, various architectures have been developed to improve this energy absorption. One of the most promising designs, based on the dumbbell shape [4,5,6], is shown in Figure 1.

However, in developing of nanostructures with novel functionalities, the use of self-locking strategies is still limited to certain niches [7,8]. For example, self-assembled molecular structures are sometimes designed to be self-locking [9]. It would be obvious to extend this concept to other areas of nanoscience, such as the broad field of 2D materials. At the nanoscale, the dumbbell shape can be easily found, for example, as a cross-section of a collapsed CNT [10], which have been mostly studied for their remarkable mechanical [11,12,13,14,15] and thermal [16,17] properties.

**Figure 2:**
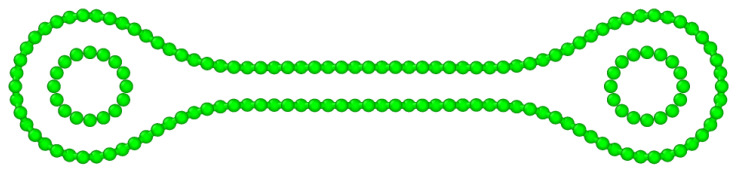
Cross-sectional view of a collapsed (60,0) CNT with (8,0) supporting CNTs.

Following this route, self-locking materials based on collapsed CNTs could be developed. While a stack of longitudinally aligned collapsed CNTs could be self-locking under low pressure, we could improve the self-locking behaviour by designing the unit block as a large collapsed CNT with thinner CNTs inserted (or grown) in both lobes, as shown in Figure 2. Indeed, thin CNTs exhibit extremely high mechanical properties (i.e., a strength of ≈100 GPa and a Young’s modulus of ≈1 TPa for the (5,5) CNTs [18]). The choice of the ratio between the radius of the outer nanotubes and that of the two supporting CNTs should enable the stable collapse of the central part of the large CNT. If the radius of the inner nanotubes is *R* and D=0.335 nm is the interlayer spacing of graphite, the outer one should have a circumference of at least C=2(2π(R+D)) for geometric constraints. In the case of self-collapse, a longer C would be needed [12]. We note that these structures are far from hypothetical, as they were obtained at the experimental level [19].

A second, stricter lower limit that could be introduced for the circumference of the outer nanotube is C=2(2π(R+D))+4(R+D). This limit would be required for a pair of circular lobes of another collapsed tube to lie in the straight part between the two circular lobes of the collapsed CNT under consideration. This condition may not be necessary to achieve the self-locking behaviour of such nanostructures. For example, a stack similar to the (50,0)–(14,0), the one built with inner (14,0) CNTs and outer (50,0) CNT, which, as we will see, is characterised by looser bonds and for which we observe stabilisation at least in the first part of the compression regime, could exhibit self-locking behaviour even though the circumference of the outer nanotube is shorter than this limit. This self-locking building block could be further stabilised by inducing defects and chemical bonds between the inner CNTs and the larger collapsed CNTs. Similarly, bonding could be implemented within the entire stack of building blocks. We note that the methods for realising these connections at the nanoscale differ from those at the macroscale and could be profitably coupled with the concept of self-locking. There are various approaches in this respect, such as electron [20] or ion beam irradiation [21,22]. A more recent approach is based on a combination of pulsed electric current, temperature, and pressure [15]. In addition, the interposition of linkers between the CNTs [23,24] could extend the applications of self-locking to chemically stabilised structures.

In the present work, we implement the idea of self-locking at the nanoscale with CNTs of different sizes and determine the mechanical properties of self-locking structures based on collapsed CNTs with lobes supported by thin CNTs. We used molecular dynamics (MD) simulations to track the atomic trajectories under compression and calculate the stress–strain curves and other mechanical properties to determine the dimensions for an optimal design of self-locking architectures. MD simulations with appropriate force fields capable of accurately tracking the high-strain regimes have indeed provided useful insights into the mechanical and thermal properties of CNTs and graphene-based structures [22,24,25,26]. However, unlike the standard evaluation of the mechanical properties of CNT bundles [27,28,29,30], where most tests are performed with loads in the longitudinal direction, that is, along the bundle axis, in this work, we focused on the mechanical properties under loading in the transverse direction, i.e., in the direction in which the nanotubes are collapsed. We find that the use of different building blocks, as determined by both the absolute value and the ratio between the outer and inner CNT radius, leads to a significantly different behaviour in the stress–strain curves and plays an important role in the self-locking mechanism. In particular, some combinations of outer and inner tubes actually show a complex collective behaviour under compression, which calls for further research on this topic.

## 2. Results

Our building blocks were designed by assembling collapsed CNTs with thin CNTs inside the lobes as support. An illustration of such a building block can be found in Figure 2.

**Figure 3:**
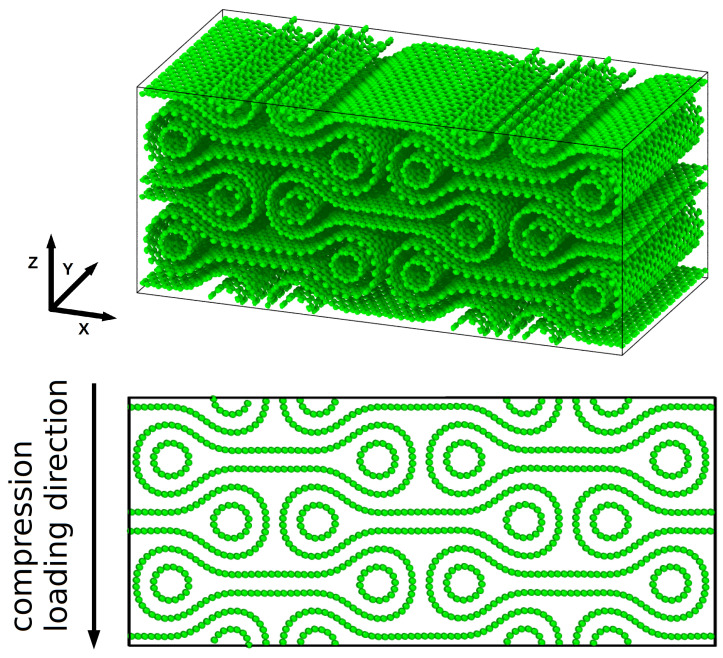
The upper panel shows a 3D view of a periodic stack of a collapsed (50,0) CNT with (8,0) supporting CNT which was relaxed using molecular dynamics. The bottom panel shows a cross-sectional view of the same stack along the direction of compressive strain (Z axis).

First, we investigated the combination of collapsed (50,0), (60,0), (70,0), and (80,0) zig-zag CNTs with (8,0), (10,0), (12,0), and (14,0) CNTs inside the lobes. In the case of the largest collapsed CNT, we used the (16,0) and the (18,0) instead of the (8,0) as supporting CNTs. The choice to focus on zig-zag CNTs is not particularly limiting, but this study could be extended to the different combinations of CNT chiralities (despite the fact that zig-zag CNTs are simply the most stable at long length [31], chirality does not matter in this context). The building blocks were arranged as in Figure 1, which on the nanoscale leads to the arrangement shown in Figure 3 for the case of a (50,0)–(8,0) stack. The simulation cell consists of a total of 8 blocks in the plane, and periodic boundary conditions apply in the 3 orthogonal directions with 3 unitary cells that are periodically repeated in the out-of-plane direction. In Figure 3 (top panel), the calculation cell has been replicated in the Y direction for the sake of clarity. Most of the information can be derived from analysing the cross-sectional view, so that we will limit ourselves to this in the following analysis.

**Figure 4:**
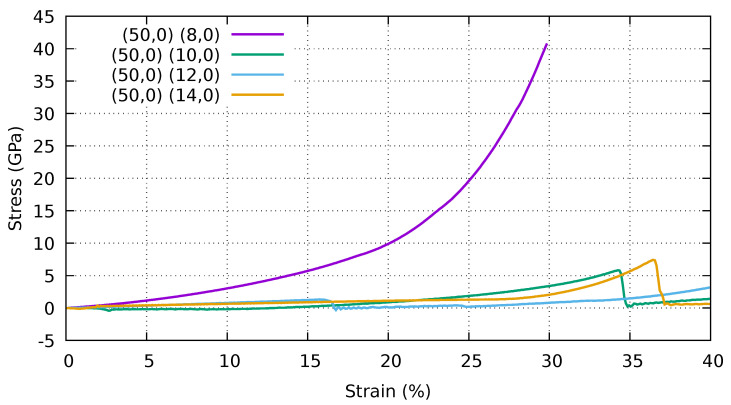
Stress–strain curves of a stack of collapsed (50,0) CNTs and various supporting CNTs ((8,0), (10,0), (12,0), (14,0)). Periodic boundary conditions were applied to the simulation cell in all directions. Stress and strain refer to the components along the Z (vertical) direction.

In Figure 4, we report the stress–strain curves of a periodic stack of collapsed (50,0) CNTs and various supporting CNTs ((8,0), (10,0), (12,0), (14,0)).

According to our simulations, the mechanical failure of these systems can be explained in two ways. The first type of mechanical failure occurs when the structure is strongly self-locking and the failure is caused by a single component. In this case, we plotted the stress–strain curve only up to the maximum stress before mechanical failure. The second type of failure is due to sliding between different building blocks in the direction perpendicular to the direction in which the pressure is applied. In this case, we plot the entire stress–strain curve as the energy release is limited and the simulations are still stable after unlocking.

**Figure 5:**
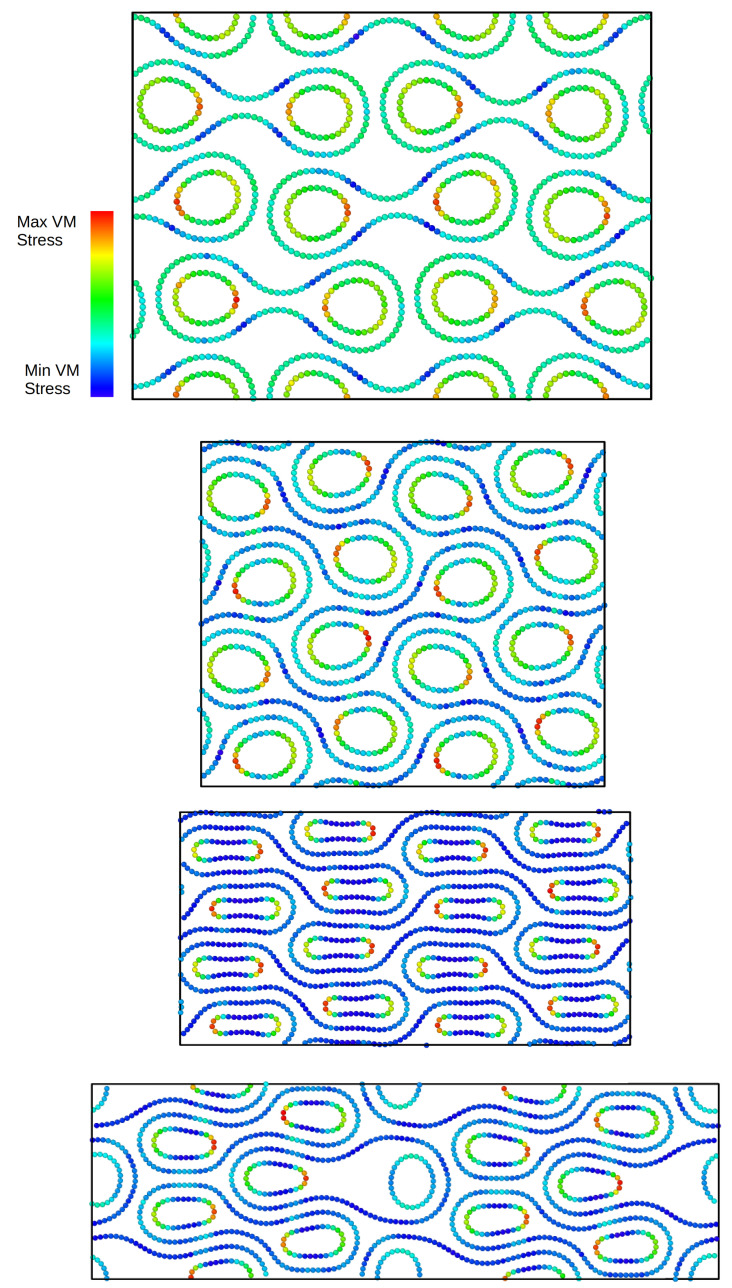
Snapshots of the atomic configurations for the (50,0)–(14,0) stack at different degrees of strain. The upper panel shows the almost symmetric relaxed configuration. The second panel from the top shows the asymmetric configuration at 10% strain. The configuration at 30% strain is shown in the third panel. Finally, the configuration at 40% strain is shown in the bottom panel.

As one can see, there is a significant difference between the behaviour of the stack when using the inner CNTs (8,0) with the smallest diameter and the other cases. In this case, the self-locking is very efficient and the system is stable under compression, allowing a progressive increase in stress during compression, which corresponds to a stiffening of the material. The failure of the system is caused by a mechanical failure of the single building block. In such a case, we plotted the stress–strain curve at this specific maximum strain before mechanical failure. In the second case (50,0)–(10,0), the stress initially decreases and becomes negative due to the equilibrium between the elastic energy and the interlayer energy. The stress then tends to increase. At 34% strain, the stack collapses. We note that the scenario for the (50,0)–(12,0) stack is somewhat complementary to that of the sample built with (50,0)–(10,0) CNTs. Indeed, we reached a stable minimum during the initial relaxation, but the structure loses self-locking at relatively low strain (17%). For the structure with the larger inner CNTs (50,0)–(14,0), the structure initially rearranges, followed by a slow increase in stress, and the stack loses self-locking at 37% strain. Four snapshots of the atomic configurations for the (50,0)–(14,0) stack at different degrees of strain are shown in Figure 5. The upper panel shows the almost symmetrical relaxed configuration. Even if this configuration is locally stable, it is unstable under compression. The second panel below shows the asymmetric configuration at 20% strain, where the structure is more compact. In the third panel from the top, you can see the still asymmetric configuration at a strain of 30%, and in the bottom panel, the configuration at a strain of 40% where some of the lobes are no longer unlocked.

**Figure 6:**
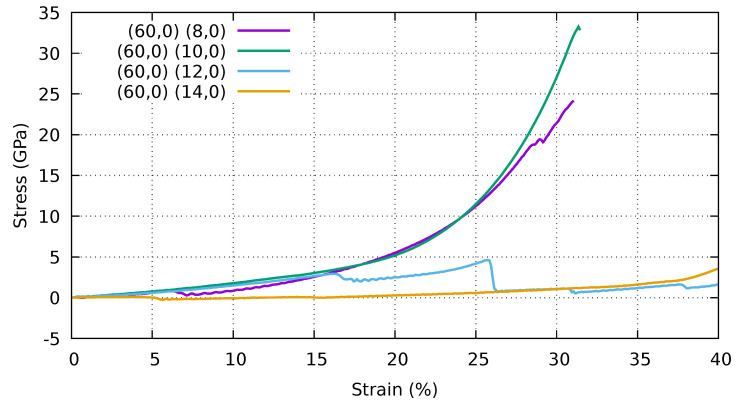
Stress–strain curves of a stack of collapsed (60,0) CNTs with different support CNTs ((8,0), (10,0), (12,0), (14,0)). Periodic boundary conditions are applied to the simulation cell in all directions. Stress and strain refer to the components along the Z (vertical) direction.

**Figure 7:**
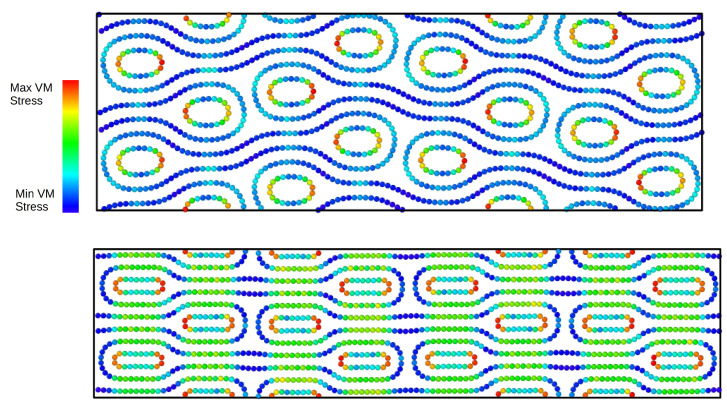
Snapshots of the atomic configurations for the (60,0)–(10,0) stack at different degrees of strain. The top panel shows the scaled snapshot for the asymmetric configuration at 10% strain and the bottom panel shows the symmetric configuration at 30% strain.

In Figure 6, we show the stress–strain curves of a periodic stack of collapsed (60,0) CNTs and various supporting CNTs ((8,0), (10,0), (12,0), (14,0)). For the (60,0)–(8,0) sample, we have a small rearrangement that brings the self-locking elements into an asymmetric configuration, which, however, seems to be quite stable. Similarly, the stack (60,0)–(10,0) is stable and goes from an asymmetric self-locking configuration to a symmetric one. We showed this transition in Figure 7. The structure consisting of (60,0)–(12,0)-CNTs is again asymmetric and loses self-locking at 16% strain. For (60,0)–(14,0), we again obtain an asymmetric structure in the direction of compression. The stress increases evenly up to 40% strain.

**Figure 8:**
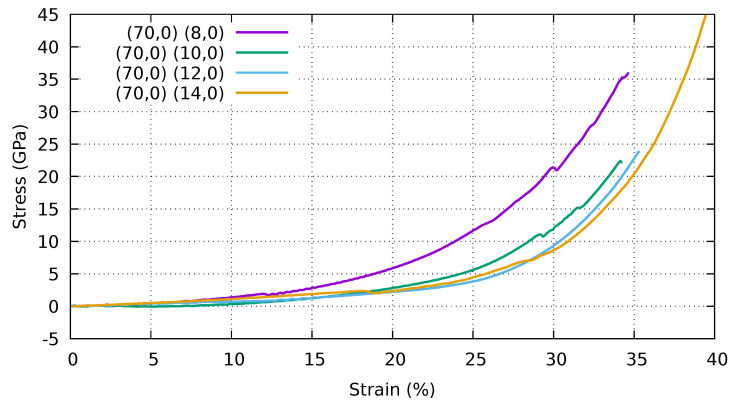
Stress–strain curves of a stack of collapsed (70,0) CNTs and various supporting CNTs ((8,0), (10,0), (12,0), (14,0)). Periodic boundary conditions are applied in all directions. Stress and strain refer to the components along the Z (vertical) direction.

In Figure 8, we show the stress–strain curves of a periodic stack of collapsed (70,0) CNTs and various supporting CNTs ((8,0), (10,0), (12,0), (14,0)). In the sample (70,0)–(8,0), as in the previous case, we have a small rearrangement that brings the self-locking elements into an asymmetric configuration. This configuration is stable up to 12% strain when another small rearrangement occurs. The new configuration is still self-locking until the stack fails at 35% strain. The (70,0)–(10,0) specimen is self-locking with an asymmetrical arrangement and can gradually bear the load up to a strain of 34%. The (70,0)–(12,0) has a similar behaviour to the (70,0)–(10,0). The (70,0)–(14,0) stack is self-locked with a small asymmetry in arrangement, its deformation under compression, and the maximum stress, are higher than those of the other samples. This can be explained by larger inner nanotubes and the symmetrisation of the structure at high pressure, respectively.

**Figure 9:**
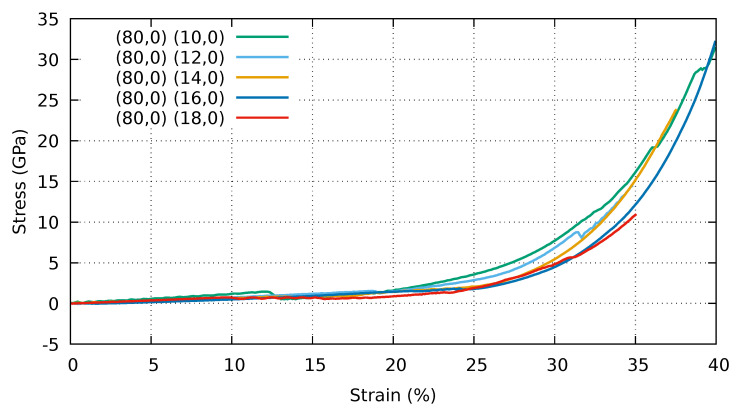
Stress–strain curves of a stack of collapsed (80,0) CNTs and various supporting CNTs ((10,0), (12,0), (14,0), (16,0), (18,0)). Periodic boundary conditions are applied to the simulation cell in all directions. Stress and strain refer to the components along the Z (vertical) direction.

**Figure 10:**
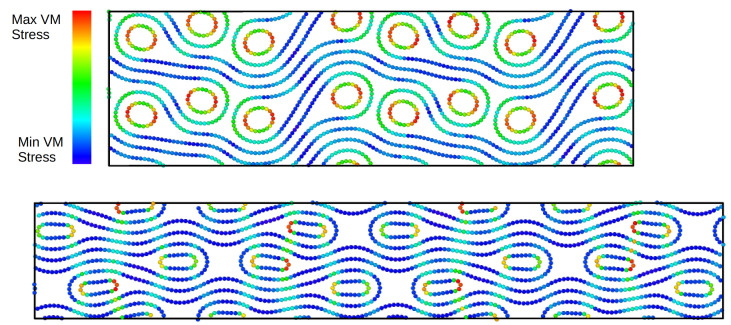
Snapshots of the atomic configuration for the (80,0)–(10,0) stack at different degrees of strain. The top panel shows the snapshot for the asymmetric configuration at 10% strain and the bottom panel shows the still asymmetric configuration at 30% strain.

In Figure 9, we show the stress–strain curves of a periodic stack of collapsed (80,0) CNTs and different supporting CNTs ((10,0), (12,0), (14,0), (16,0), (18,0)). For the sample (80,0)–(10,0), there is a linear increase in stress that brings the system into a different asymmetric configuration at 12.5% strain. Beyond this point, the system is self-locking and stable. Figure 10 shows two snapshots of the atomic configurations for the (80,0)–(10,0) stack at different degrees of strain. The top panel shows the snapshot for the asymmetric configuration at 10% strain and the bottom panel reports the snapshot for 30% strain. It is worth noting that, in the case of 30% strain, a horizontal separation occurs between the lobes of the building blocks on the same plane. For the samples (80,0)–(12,0) and (80,0)–(14,0), we show interrupted stress–strain curves since the systems exhibit mechanical failure. In contrast, the strain is not sufficient to cause the stacks (80,0)–(16,0) to fail, although the behaviour is similar to the previous cases. In the last case, (80,0)–(18,0), we again obtained a failure under compression.

We note that, for the (80,0) CNT, all stacks are locked, so the difference between the samples is whether they break under compression or not. We see that some configurations fail due to fractures of the inner nanotubes, which are the most deformed structures. If the structural arrangement is such that the stress is concentrated on the edge of the inner nanotubes, the stack will break as soon as it is compressed. In the optimal cases, the stress is distributed over the stack and not concentrated on these points. We hypothesise that for longer nanotubes, more than one optimal alignment of the inner and outer nanotubes can be found, as the stress is not concentrated on the edges of the inner nanotubes. The investigation of the failure mechanisms of self-locking stacks at high pressure should be performed with ad hoc simulations.

In Table 1, we summarise the fracture or unlocking stresses. We summarise the fracture or unlocking strains in Table 2.

In Table 3, we give the calculated SAE (see Equation (4) for the stacks up to a strain of 40%. For those where there is an abrupt failure of the individual building block and no unlocking, the SAE is given up to the value of the failure strain.

We further extended the analysis at a temperature of 300 K in order to verify the possible influence of this parameter. Overall, we found that the distinction between self-locking and not self-locking stacks is very similar to that obtained at 1 K. For the stacks built with smaller collapsed nanotubes, a higher temperature can open the collapsed nanotubes; however, stacks built with larger nanotubes are stable and the influence of the temperature is small. We found that all the stacks that are self-locked at 1 K are also self-locked at 300 K.

## 3. Discussion

As the results show, the behaviour of the collapsed CNT stacks under compression strongly depends on the relative size of the inner and outer CNTs. In addition, the absolute dimensions of the CNTs play a role, so that, for example, space is created between the lobes during compression, as in the (80,0)–(10,0) stack. The failure of the system can occur due to the abrupt release of potential energy stored in the system or due to a softer unlocking of the building blocks due to a slip in the direction transverse to the direction of compression. We have found that there are different regimes under compression. If there is no spatial gap between the building blocks and the structure is symmetrical, the compression is uniform in the direction of self-locking. If the initial configuration is symmetric but shows some spatial gaps between the collapsed CNTs, the structure leans towards an asymmetric state. In addition, some stacks are asymmetric from the beginning but tend to be symmetric under high compressive loading. Finally, in the case of large outer collapsed CNTs and thin inner CNTs, we found that some space is created between the lobes of different collapsed CNTs on the same plane. In general, the key observable associated with the loss of self-locking is the sudden expansion of the cell in the X direction (in our images horizontal, perpendicular to both the compression direction and the axis of the nanotubes). However, it is not obvious whether a certain degree of asymmetry can characterise the loss of self-locking, since asymmetric configurations can also be self-locking and unlocking is a dynamic process. For high-throughput screening, it is probably safer to rely on the stress–strain curves and signs of the abrupt expansion of the cell to determine the loss of self-locking. As for the self-locking properties and symmetry, we summarise the data in Table 4 and Table 5.

As for the shape of the stress–strain curves, the slope of the curves increases with increasing compression, which corresponds to a stiffening of the material under compression. We note that this is different from, for example, the compression of 3D structures such as carbon nanotruss networks [25] and carbon nanofoams [26]. In these cases, the stress–strain curve initially shows a steep increase, followed by a plateau during the collapse of the structure, and finally, a steep increase in stress when the structure is fully compressed.

We also note some differences with the macroscopic case of, for example, steel or PLA dumbbell tubes [4,6]. In the macroscopic case for the dumbbell-shaped tube, the angle between the round and the flat part (Figure 1) tends to remain constant under compression, as the steel is shaped with this angle from the beginning and the tube opens in the centre, effectively assuming a three-lobed shape. In the case of our systems, the bending stiffness of graphene is low and there is no similar effect. In addition, the stress–strain curves for the self-locking stacks are almost linear for the structures realised at the macroscopic level with dumbbell-shaped tubes [4,6], while they are non-linear for our samples and show stiffening under compression. We note that the linearity of the stress–strain curve at the macroscopic level could be due to the design. In fact, similar results were obtained for steel and PLA structures [4,6].

## 4. Materials and Methods

The mechanical properties were calculated using the MD method implemented in the LAMMPS code [32]. The calculations were performed with the interatomic potential AIREBO [33].

The AIREBO potential energy consists of three terms

(1) $$E=\frac{1}{2}\sum_{i}\sum_{j\neq i}\left[E_{ij}^{\mathrm{REBO}}+E_{ij}^{\mathrm{LJ}}+\sum_{k\neq i,j}\sum_{l\neq i,j,k}E_{kijl}^{\mathrm{TORSION}}\right],$$

where *i*, *j*, *k*, *l* denote the atoms. The first term is the Reactive Empirical Bond Order (REBO) potential [34], the second is the Lennard–Jones term for the non-bonded interaction, and the third is an explicit 4-body potential for the description of the dihedral angles.

The temperature was set to T=1 K and the pressure control in the directions transverse to the deformation was realised with the Nosé–Hoover barostat–thermostat damping coefficient, which was assumed to be 1 in our simulations. The lateral pressure was set to a target value of 0 Pa to simulate free boundaries. The velocity Verlet integrator with a time step of 1 fs enables the correct integration of Newton’s equations of motion (i.e., the conservation of the total energy). Prior to the mechanical tests, all samples were fully equilibrated at T=1 K and a pressure of zero.

The simulations were performed by forcing a uniform contraction of the simulation cell in the vertical direction and remapping the atomic positions (see the Figure 5, Figure 7 and Figure 10).

We evaluated the mechanical properties by calculating the total stress of the system, and in particular, the component of the total stress along the direction of compression. The strain parallel to the direction of deformation is defined by:

(2) $$\varepsilon =\frac{L-L_{0}}{L}=\frac{\Delta L}{L},$$

where $L_{0}$ and *L* are the initial and actual length of the sample in the direction of loading.

The uniaxial compressive load was applied until a total strain of 40% was reached. The applied strain rate was set to 0.1% per ps. Stress and strain were stored every 1000 time steps.

To determine the stress, the components of the compressive stress tensor in response to the external deformation are calculated as [35]

(3) $$P_{ij}=\frac{\sum_{k}^{N}m_{k}v_{k_{i}}v_{k_{j}}}{V}+\frac{\sum_{k}^{N}r_{k_{i}}f_{k_{j}}}{V},$$

where *i* and *j* denote the coordinates *x*, *y*, *z*; *k* runs over the atoms; $r_{k_{i}}$, $m_{k}$, and $v_{k}$ are the position, mass, and velocity of the *k*th atom; $f_{k_{j}}$ is the *j*th component of the total force on the *k*th atom due to the other atoms; and finally, *V* is the volume of the simulation box.

The performance of our stacks as energy absorbers was assessed by calculating the specific energy absorption (SAE):

(4) $$\mathrm{SAE}=\frac{E_{t}}{M},$$

where *M* is the mass of the sample and $E_{t}$ is the total absorbed energy defined as follows:

(5) $$E_{t}=V\int_{0}^{\varepsilon }\sigma \left(\varepsilon^{\prime }\right)d\varepsilon^{\prime },$$

where $\sigma (\varepsilon^{\prime })$ is the stress at $\varepsilon^{\prime }$ strain, and *V* is the sample volume. The upper integration limit was set at 40% strain or the strain at which the stacks fail abruptly.

Snapshots of the atomic configurations were taken with the OVITO package [36]. The von Mises stress was calculated as follows

(6) $$\sigma_{\mathrm{VM}}=\sqrt{\frac{1}{2}\left[{\left(\sigma_{11}-\sigma_{22}\right)}^{2}+{\left(\sigma_{22}-\sigma_{33}\right)}^{2}+{\left(\sigma_{33}-\sigma_{11}\right)}^{2}\right]+3\left(\tau_{12}^{2}+\tau_{23}^{2}+\tau_{31}^{2}\right)},$$

where σ and τ are the normal and the shear stresses, respectively. The von Mises stress was visualised using the same colour code for the atomic configurations at different stages of compression, i.e., blue (minimum stress) and red (maximum stress).

We note that there is indeed a difference between $\sigma_{i,j}$ and $\tau_{i,j}$ used in Equation (6) and $P_{i,j}$ used in Equation (3). The $P_{i,j}$ components in Equation (3) are the components of the stress tensor of the entire calculation cell. The quantities $\sigma_{i,j}$ and $\tau_{i,j}$ used in Equation (6), on the other hand, are quantities per atom, i.e., they are the components of the stress on each individual atom.

## 5. Conclusions

In this work, we used molecular dynamics to investigate the mechanical properties of self-locking stacks based on collapsed CNTs. We have shown that the self-locking properties can be improved by inserting thinner CNTs into the lobes of the collapsed CNTs.

We analysed in detail the atomic configurations of the structures corresponding to the different regimes shown in the stress–strain curves. Our study shows that the building blocks used in the present work lead to a complex collective behaviour. For certain combinations of the dimensions of the outer and inner CNTs, we obtained self-locking stacks. The ratio between the outer and inner CNT dimensions plays a role in self-locking, as does the absolute dimension of the same components.

In general, we found that self-locking can be achieved in most of the stacks analysed. However, in only a few cases, we found self-locking without rearrangement with respect to the relaxed initial configuration. In particular, the combinations of CNTs that provided us with the smoother stress–strain curves were (50,0)–(8,0), (60,0)–(10,0) and (70,0)–(10,0). Applications that could utilise the self-locking mechanism of these nanostructures under pressure are conceivable in electronics [37], thermodynamics [16], and mechanics [15]. For example, the electronic properties can be tuned by applying pressure, whereby a mechanically stable structure is maintained even without chemical contact between the collapsed tubes. In thermal applications, the ultra-high thermal conductivities of the individual CNTs are maintained during the collapse [16], making these self-locking stacks suitable for fibres with high thermal conductivity and high mechanical stability. In addition, the thermal conductivity can be adjusted in the transverse direction by applying a load to the stack [25,38]. The self-locking would be also a useful way to have nanotube cables resistant to transverse separation. We also devise that self-locking mechanisms could be widely applied in 2D-layered materials, opening up new possibilities for developing advanced materials and devices.

Finally, future prospects and further investigations could consider, e.g., increasing the size of the building blocks by using multilayer CNTs or connecting the nanotubes by, e.g., single-carbon atom linkers. On the computational side, different force fields could be used to study specific regimes. For example, further insights could be gained by using the AIREBO-M [39] force field, which is valid for high-pressure regimes, or by replacing the long-range interaction with the potential to better treat the interactions between the layers, such as the dihedral-angle-corrected registry-dependent potential (DRIP) [40], which can also be coupled with potentials that can treat bonded interactions, such as REBO [33] or Tersoff [41].

## Figures and Tables

**Table 1:** Fracture or unlocking stress for the various stacks.

		Inner					
		**(8,0)**	**(10,0)**	**(12,0)**	**(14,0)**	**(16,0)**	**(18,0)**
outer	(50,0)	40.2	5.83	0.73	7.43	-	-
	(60,0)	24.2	33.3	2.92	>3.56	-	-
	(70,0)	36.0	23.4	23.7	44.9	-	-
	(80,0)	-	>31.5	14.4	22.5	>32.3	10.9

**Table 2:** Fracture or unlocking percentage strain for the various stacks.

		Inner					
		**(8,0)**	**(10,0)**	**(12,0)**	**(14,0)**	**(16,0)**	**(18,0)**
outer	(50,0)	29.8	34.3	16.6	36.5	-	-
	(60,0)	31.1	31.4	16.3	>40.0	-	-
	(70,0)	34.6	34.2	35.3	39.4	-	-
	(80,0)	-	>40.0	34.8	37.2	>40.0	35.1

**Table 3:** The values of the SAE in MJ Kg^−1^ are given for the various stacks.

		Inner					
		**(8,0)**	**(10,0)**	**(12,0)**	**(14,0)**	**(16,0)**	**(18,0)**
outer	(50,0)	2.74	0.27	0.20	0.38	-	-
	(60,0)	1.09	1.31	0.43	>0.16	-	-
	(70,0)	2.05	1.14	1.35	2.28	-	-
	(80,0)	-	>1.81	0.73	0.96	>1.19	0.57

**Table 4:** The property of being self-locking (L) until abrupt failure (at 40% strain) or unstable and unlocking (U) is summarised for the different stacks.

		Inner					
		**(8,0)**	**(10,0)**	**(12,0)**	**(14,0)**	**(16,0)**	**(18,0)**
outer	(50,0)	L	U	U	U	-	-
	(60,0)	L	L	U	L	-	-
	(70,0)	L	L	L	L	-	-
	(80,0)	-	L	L	L	L	L

**Table 5:** The property of being symmetrical (S) or asymmetrical (A) in relation to the compression axis is summarised for the different stacks. The arrows indicate the cases in which the symmetry has changed due to the compression.

		Inner					
		**(8,0)**	**(10,0)**	**(12,0)**	**(14,0)**	**(16,0)**	**(18,0)**
outer	(50,0)	S	A	A	A	-	-
	(60,0)	A	A → S	A	A	-	-
	(70,0)	A	A	A	A → S	-	-
	(80,0)	-	A	A	A	A → S	A

## Data Availability

Data is contained within the article.
